# The joints in juvenile idiopathic arthritis

**DOI:** 10.1007/s13244-015-0406-0

**Published:** 2015-04-23

**Authors:** Lil-Sofie Ording Muller, Paul Humphries, Karen Rosendahl

**Affiliations:** Department for Radiology and Intervention, Oslo University Hospital, Oslo, Norway; Institute of Child Health, UCL, University College London Hospital NHS Trust and Great Ormond Street Hospital for Children, London, UK; Department of Radiology, Haukeland University Hospital, Bergen, Norway

**Keywords:** Juvenile idiopathic arthritis, Magnetic resonance imaging, Ultrasound, Paediatrics, Musculoskeletal

## Abstract

**Abstract:**

Juvenile idiopathic arthritis is the most common rheumatic entity in childhood. Imaging has become an important supplement to the clinical assessment of children with JIA. Radiographs still play an important role in the workup, and long-term follow-up in children with JIA, but are not sensitive to findings in the early disease stage. Both ultrasound and MRI are more sensitive to inflammatory changes than clinical assessment alone. However, the differentiation between normal findings and pathology can be challenging, particularly in early disease. The objective of this review is to discuss the role of imaging in JIA, describe the typical findings on different modalities and highlight the challenges we face regarding the reliability and accuracy of the different methods for imaging the joints in children with JIA.

***Key Points*:**

• *Imaging is an important supplement to the clinical examination in JIA*.

• *Ultrasound is more sensitive for detecting synovitis than clinical examination alone*.

• *MRI can depict all relevant structures in joint inflammation*.

• *The differentiation between normal variants and pathology is difficult in children*.

## Introduction

Juvenile idiopathic arthritis (JIA) is defined as arthritis of unknown cause, with disease duration of more than 6 months, occurring in children under 16 years. It is the most common rheumatic entity in childhood with a prevalence of 0.6–1.9 in 1000 children. The exact pathogenesis is not fully understood but is thought to include both genetic and environmental components. JIA is not one single disease but includes a subset of different childhood arthritides (Table 1). Both clinical presentation and outcome vary with clinical subtype, and persistent oligoarthritis has been shown to have the best prognosis [1–3].

**Table 1:** Summary of the main features of the six different subtypes of JIA

Subset of JIA	Frequency %	Age at onset	Clinical presentation	Sex
Oligo	27–56	Early childhood, peak 2–4 years	Four or fewer joints involved the first 6 months	F> > M
Poly RF negative	11–28	Early peak 2–4; late peak 6–12 years	Four or more joints involved within the first 6 months, absence of IgM RF. Heterogeneous disease with three subsets. Prognosis varies with the disease subset	
Poly RF positive	2–7	Late childhood-adolescence	Four or more joints involved within the first 6 months, IgM RF positive. Resembles adult RA. Involvement of small joints. Progressive and diffuse joint involvement	F> > M
Ethesitis related	3–11	Late childhood-adolescence	Characterised by enthesitis and arthritis. Often HLA-B27 positive. Commonly hip involvement at presentation. Often a mild and remitting course but may progress with sacroiliac and spinal joint involvement, resembling ankylosing spondylitis	M> > F
Psoriatic	2–11	Early peak 2–4 years; late peak 9–11 years	Arthritis and psoriatic rash or psoriasis in close family. Controversial definition, resembles oligoarthritis but more often with dactylitis and involvement of both small and large joints	F > M
Systemic	4–17	Throughout childhood	Arthritis and quotidian fever plus one or more of the following symptoms: characteristic rash, hepatomegaly, splenomegaly, lymphadenopathy, serositis. Variable course; 5–8 % develop macrophage activation syndrome	F = M

Despite the heterogeneity, it is likely that there is some genetic overlap, as all JIA subtypes share joint inflammation as the most prominent disease feature [1–4].

Joint pathogenesis involves inflammation of the synovial lining, with the potential to cause joint destruction. There is infiltration of the synovium by inflammatory cells. The lining layers of the synovium then become hyperplastic with increased vascularity. The pannus is comprised primarily of invasive cells and the synovium becomes locally invasive at the synovial interface with cartilage and bone. Subsequent destruction of the bone and cartilage occurs as a result of antibody deposition and degradative enzymes [5]. It is believed that the bone destruction in JIA is a consequence of overlying cartilage degradation. The inflammation may also cause growth disturbances, both systemically and locally in the affected joint. The typical manifestations of the articular and periarticular inflammation in early and late disease are shown in Table 2.

**Table 2:** Typical manifestations of the articular and periarticular inflammation of JIA in early and late disease. The order of presentation is not absolute; some of the features may not be present at all or the features may overlap

Periarticular soft tissue change	Early Late
Synovitis	
Tendinitis	
Bursitis	
Periarticular bony change	
Bone oedema	
Periostitis	
Growth disturbances	
Osteoporosis	
Destructive change of bone and cartillage	
Erosive change of bone	
Joint space narrowing, bone fusion, malalignment	

The objective of this review is to discuss the role of imaging in JIA, describe the typical findings on different modalities and highlight the challenges we face regarding the reliability and accuracy of the different methods for imaging the joints in children with JIA.

## The role of imaging in JIA

Currently, the diagnosis of JIA is based on clinical and laboratory findings and does not include imaging. However, clinical assessment of children with joint complaints is challenging and laboratory findings are often equivocal. This has led to an increased use of imaging for both diagnosis and follow-up, particularly of joints that are frequently affected but may be asymptomatic, such as the temporomandibular joint (TMJ) [6].

Imaging is used to determine the presence and extension of joint inflammation and can more accurately distinguish between arthritis and tenosynovitis than clinical examination alone. In some cases, it can also help define the subtype of JIA. Imaging has a role in the assessment of differential diagnosis, such as leukaemia, avascular necrosis, trauma or bone tumours, and also shows potential in the evaluation of treatment response.

There are no unifying international recommendations for imaging in JIA and both the choice of imaging modality and when and whom to refer for radiological investigations vary between centres. One of the major challenges when interpreting imaging studies is to distinguish between normal findings and pathology in the early stages of the disease, particularly on MRI. Moreover, the lack of standardised scoring systems for all modalities makes the objective evaluation of the degree of inflammation and destruction challenging. Plain radiographs of the affected joint are often not helpful to establish the diagnosis but are advised as a baseline study for longitudinal follow-up and to help narrow the list of differentials or suggest alternative diagnoses, for example an underlying bone tumour or a fracture. Ultrasound and MRI form the basis of more advanced imaging assessment in paediatric arthritis, while cone-beam computed tomography play a role in imaging of the TMJs and conventional CT in imaging of the sacro-iliac and facet joint of the cervical spine [7].

## Radiographs

**Fig. 1:**
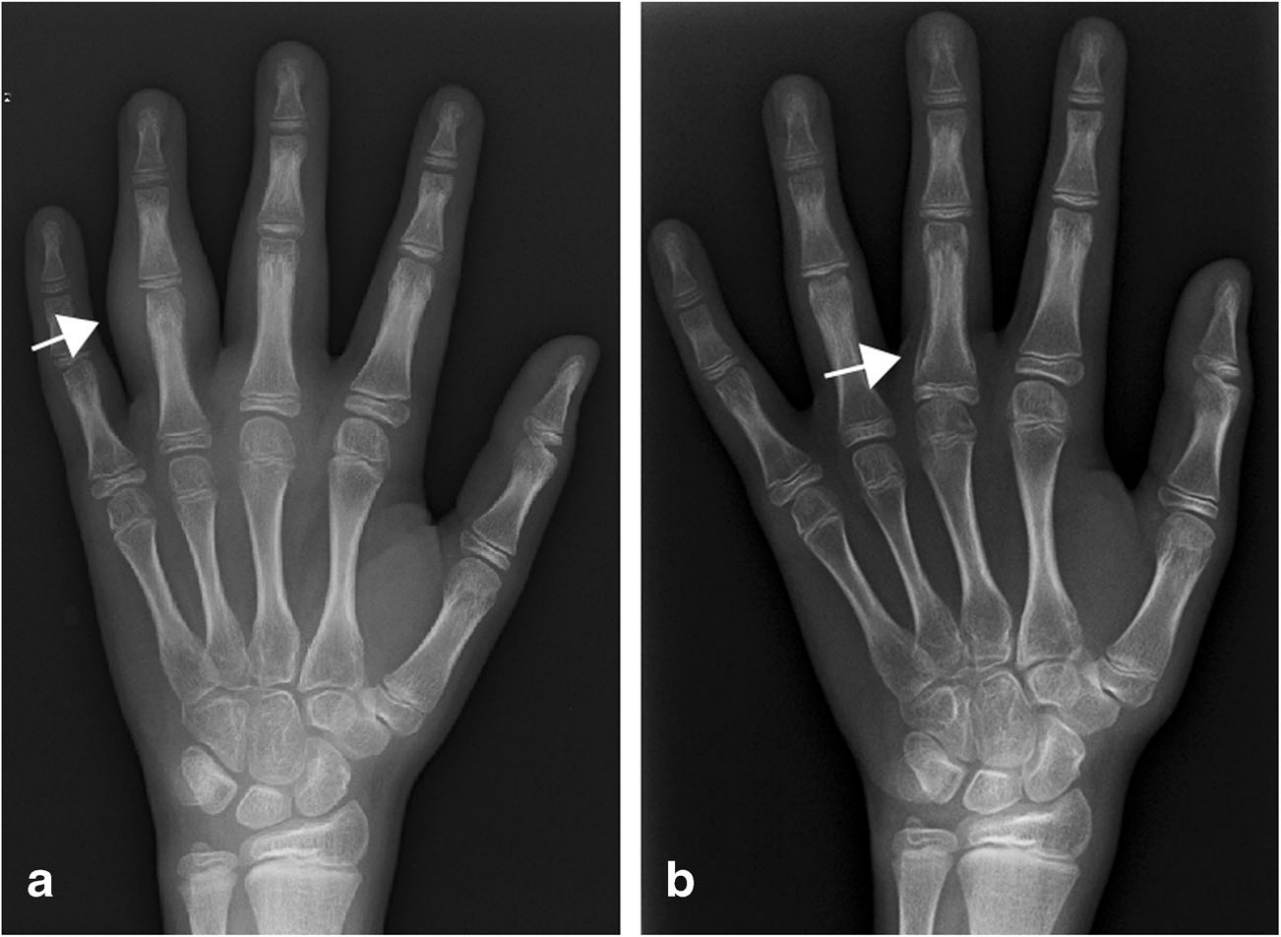
Plain radiographs of affected joints in JIA may be normal, particularly early in the disease course; however, non-specific soft tissue swelling may be seen (**a**). Periosteal reaction (**b**) may also occur in active synovitis and is most frequently seen in tenosynovitis.

**Fig. 2:**
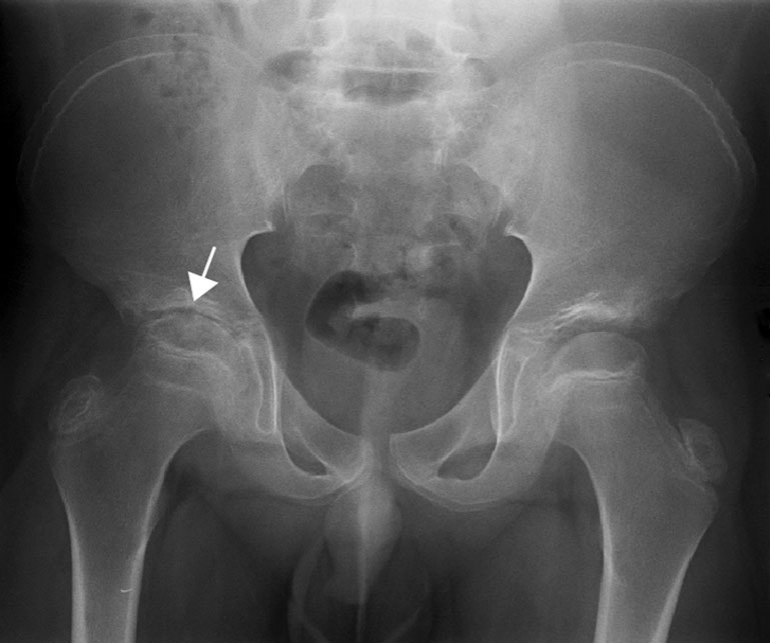
Erosions and joint space narrowing can be seen on radiographs (arrow) but are normally a late finding in arthritis

Joint malalignment, evaluation of focal growth disturbances and joint damage evaluation in JIA have traditionally been performed by X-ray scoring methods. Radiographs can show soft tissue swelling and periostitis even in the early stages of the disease (Fig. 1) but the findings are not specific for arthritis. Growth disturbances can be seen early as focal accelerated growth or advanced skeletal maturation or later as premature closure of growth plates or squaring of the bones. Radiographs may show bone erosions and may depict cartilage loss indirectly through joint space narrowing [8] (Fig. 2). Joint space narrowing, malalignment and focal concavities or lytic lesions of the bones are perceived as signs of joint destruction [9]. However, the sensitivity is low, particularly for disease in early stages, and the aforementioned features are not sensitive to subtle change over time [10, 11]. Plain radiographs cannot visualise the synovium, joint effusion, articular cartilage, bone marrow, or ligaments and tendons directly. Therefore, in cases of clinical uncertainty or where treatment may be modified on the basis of activity versus quiescent disease, additional imaging modalities may be employed.

## Ultrasound

Ultrasound has many advantages in children. It is readily available, non-ionising, dynamic and well tolerated without recourse to anaesthesia or sedation; hence, it is a frequently used radiological examination in a child with JIA. Small patient size is both an advantage and disadvantage—using modern high-resolution transducers, exquisite detail is possible but requires a meticulous approach and adaptation of scanning equipment. Small “hockey-stick” transducers can be used for small joints but also for larger joints, e.g. ankle scanning in small children. Judicious use of warm jelly, often as a stand-off for small joints, and a child-friendly environment increase the chances of success.

**Fig. 3:**
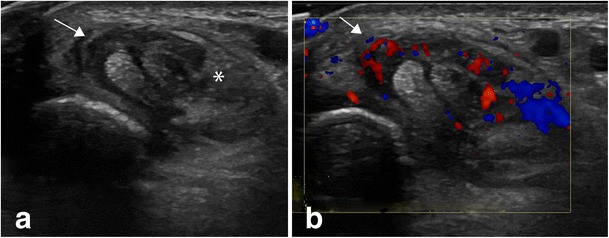
Ultrasound shows signs of tenosynovitis in the tibialis posterior and flexor digitorum longus tendons with hypertrophic synovium in the tendon sheet (**a**). The hypertrophic synovium can be both hypoechoic (arrow) and hyperechoic (asterisk). Hypervascularity is seen on colour Doppler examination (**b**)

**Fig. 4:**
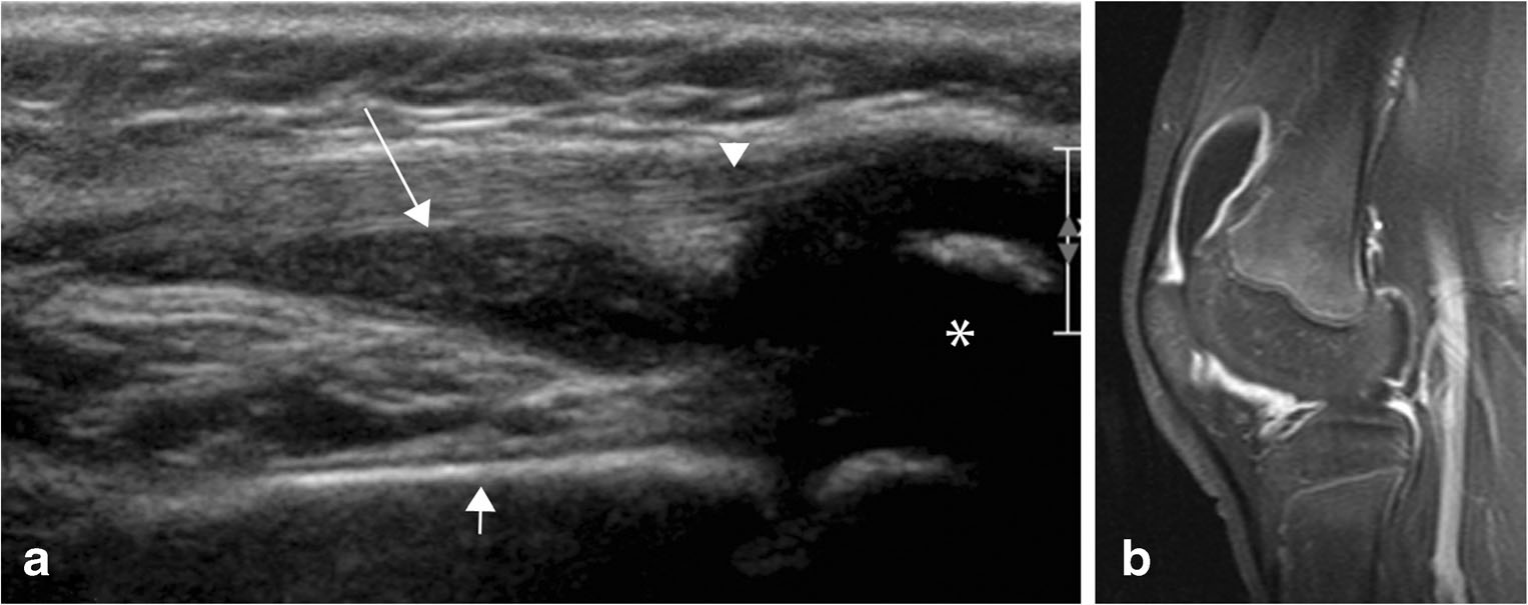
**a** Ultrasound (panoramic view) of the ventral aspect of the knee shows fluid in the supra-patellar recess (arrow), cranial to the patella (asterisk), under the patellar tendon (arrowhead) and overlying the femur (small arrow) suggestive of synovitis. **b** The findings were confirmed by MRI, which shows slightly hypertrophic, enhancing synovium in keeping with synovitis

**Fig. 5:**
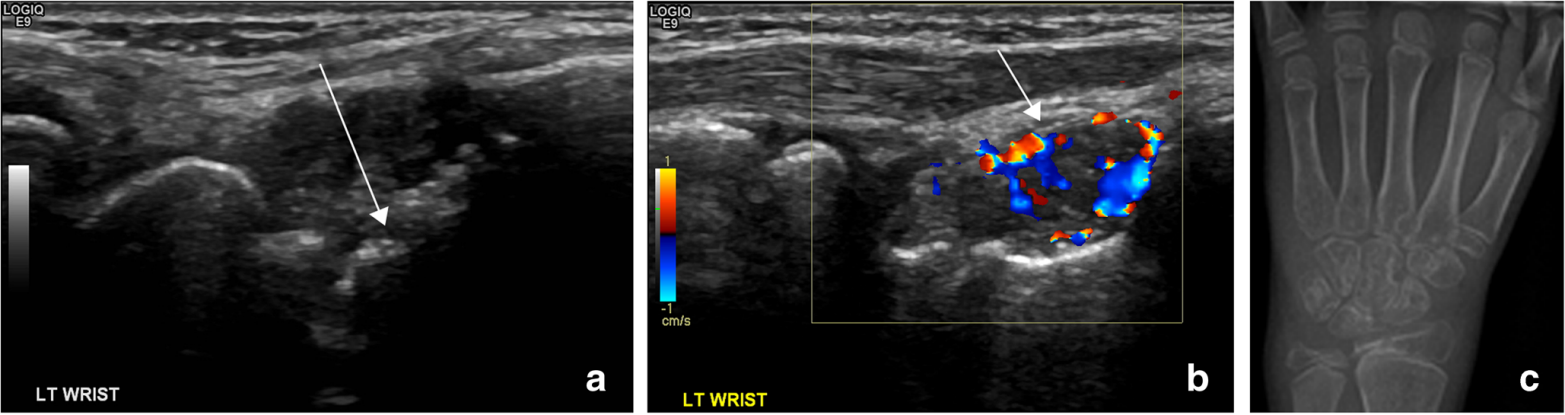
Ultrasound may depict both synovitis and bone destruction, particularly in small joints. **a** Ultrasound of the dorsal aspect of the wrist shows an irregular surface of the scaphoid (long arrow) suggestive of erosions. **c** There was thickened and hyperaemic synovium over the scaphoid, indicative of active inflammation. **c** Plain radiograph of the wrist confirmed squaring of the carpal bones, carpal crowding and a sclerotic, irregular bony surface

Diagnostic ultrasound may be used to assess for features suggesting acute or active disease, principally joint effusion, synovial thickening, teno-synovial thickening and effusion (Figs. 3, 4 and 5). Ultrasound has a higher sensitivity and specificity for the presence of synovitis than clinical assessment alone and is the most sensitive method for the detection of tenosynovitis [12, 13]. For the less experienced examiner it may be challenging to distinguish between a thickened oedematous synovium and the adjacent cartilage or joint fluid because all these structures may have a similar, hypoechoic appearance. A rule of thumb is that the joint fluid will be compressible and cartilage thickness will not change with the pressure from the ultrasound probe. The synovium will only be partly compressible and may show flow on Doppler examination. Colour and power Doppler may be utilised to assess increased vascularisation, a marker of active inflammation, to differentiate between active and quiescent disease [14]. Chronic disease changes can also be potentially assessed using ultrasound, principally cartilage reduction and bony erosions, particularly in small joints (Fig. 5). Spannow et al. documented normal changes in cartilage thickness with age and sex and described intra- and inter-observer variability of these measurements enabling a clinical assessment of cartilage thickness with reference to normal values [15]. It has been demonstrated that there is reduced cartilage thickness in patients with JIA compared to age- and sex-matched controls, although interestingly this phenomenon is observed in both clinically affected and non-affected joints [16]. The measurements obtained on US correlate with MRI measurements [15] potentially enabling longitudinal US measurements to be used for disease response assessment, with it being recognised that cartilage thickness may increase on treatment.

Ultrasound may also be used in interventional procedures, for both diagnostic purposes, i.e. aspiration of a joint, and treatment, i.e. tendon sheath or joint injection [17]. Despite the stated advantages of ultrasound, there are several limitations. Musculoskeletal ultrasound (MSK US) is not a widespread skill, particularly amongst paediatric radiologists, and there is a learning curve when undertaking these examinations. Ultrasound is an operator-dependent modality, taking time to become proficient. Close collaboration between the clinical team and radiologist, with multidisciplinary discussion where there are discordant findings between the clinical examination and the sonographic assessment, facilitates appropriate management. It is recognised that US will depict more synovitis in JIA than clinically detected [12], but this may represent old inactive changes rather than active disease. Doppler examination may be used to differentiate active from inactive disease, but there is no unifying definition of hyperaemia, and the Doppler appearance will vary with different probes, settings, vendors and the imaging technique (e.g. probe pressure). Therefore the interpretation of the findings, particularly in early disease/subtle findings, is challenging. Comparison with a contra-lateral unaffected joint or the adjacent non-inflamed synovium may help in the evaluation. Furthermore, ultrasound cannot assess changes in the bone marrow. Finally the stages of development and US imaging of the normal growing peri-articular skeletal tissues throughout childhood are in general not well documented (aside from Spannow et al. described above); hence, normal standards for US anatomy are not yet properly established.

Despite the challenges of MSK US in paediatrics there is a growing acceptance of its use and availability. US lends itself to one-stop clinics to establish a diagnosis, formulate a treatment plan and possibly US-guided aspiration or injection.

## MRI

MRI is the only diagnostic tool that can assess all relevant anatomical structures in joint inflammation. Contrast-enhanced MRI is able to depict synovial thickening and enhancement, joint fluid, bone marrow oedema as well as damage to cartilage and bone and is therefore potentially a powerful imaging tool in the assessment of joint inflammation and the progression of permanent joint damage. Erosions cannot be assessed clinically and MRI is thought to have greater sensitivity than radiography in early detection of erosions [18]. The standard MRI protocol in arthritis [19] includes a

- T1 SE sequence,
- T2 fat-suppressed sequence or a STIR and
- T1 fat-suppressed sequence pre- and post-contrast.

Some may prefer to add a sequence for visualisation of cartilage, such as a proton-weighted or 3D DESS sequence. The DIXON fat-suppression technique is increasingly being used because it gives robust, homogeneous fat suppression and can be combined with high-resolution T2-weighted images, giving a better signal-to-noise ratio compared to the STIR sequence, without increasing the scan time [20]. Contrast administration is necessary for the assessment of synovitis; hence contrast enhanced MRI is part of the standard protocol in JIA. The preferred image planes will vary depending on the joint but the MRI protocol should allow multiplanar reformatting so potential findings, particularly evidence of joint destruction, could be verified in at least two planes.

**Fig. 6:**
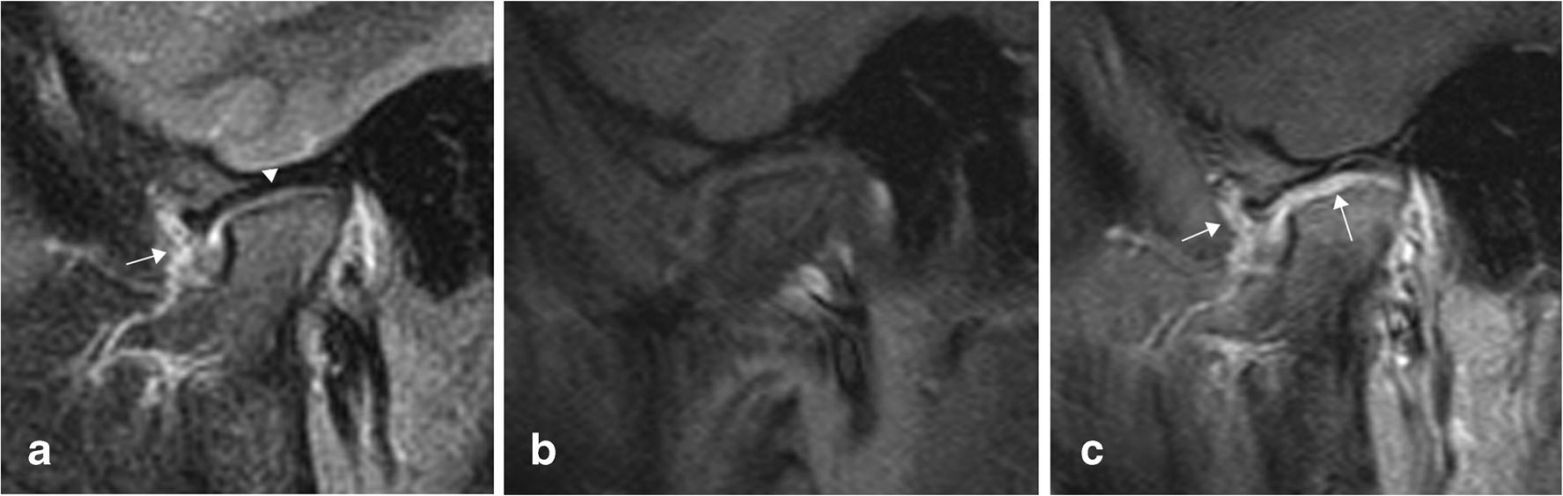
Sagittal STIR (**a**), T1 f. pre-contrast (**b**) and T1 f. post-contrast image (**c**) of the temporomandibular joint showing thickened, enhancing synovium (arrows) and a sliver of fluid within the joint (arrowhead) indicating active inflammation.

**Fig. 7:**
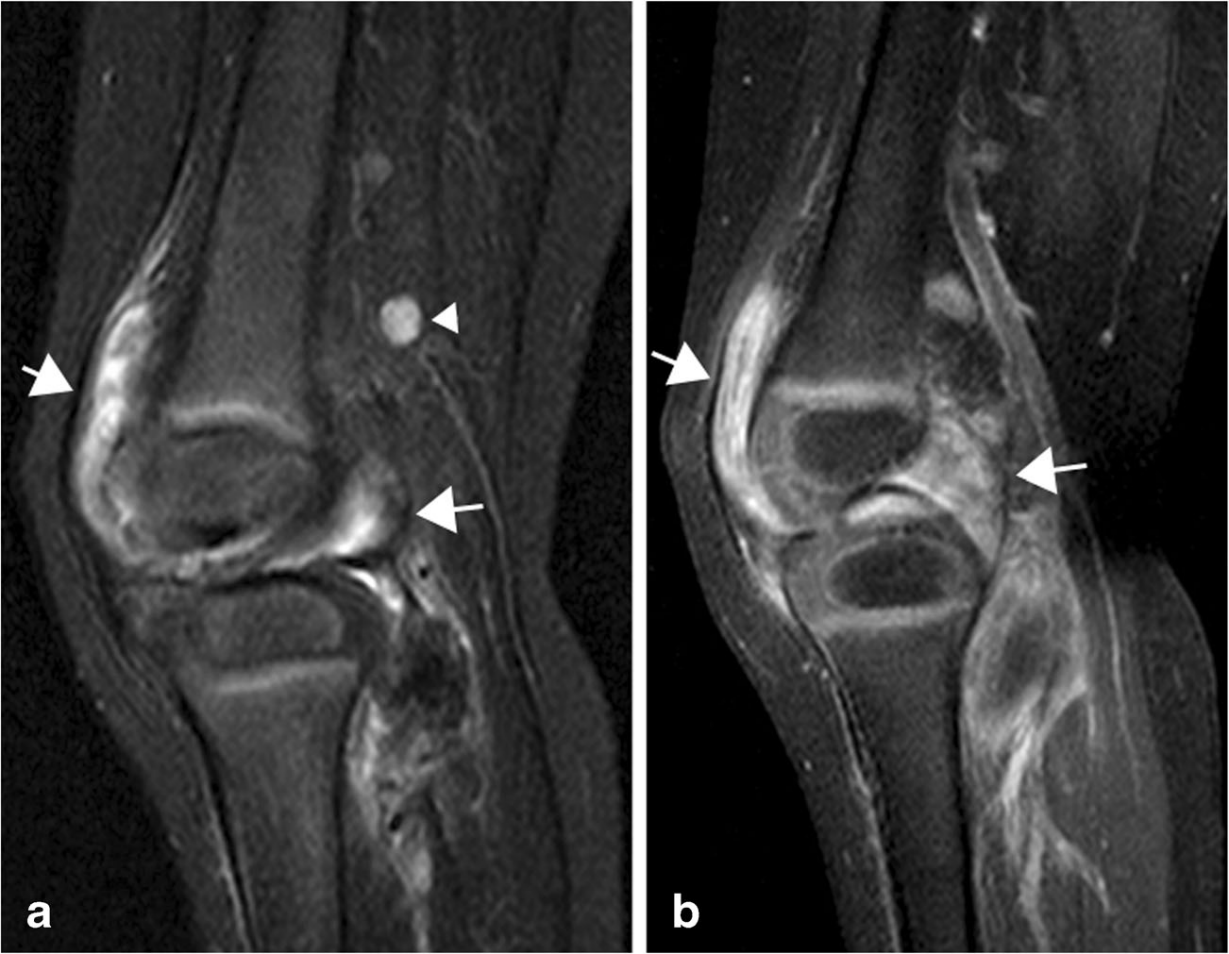
**a** Sagittal STIR sequence of a knee with long-standing arthritis showing marked synovial hypertrophy (arrows) and marked lymph nodes (arrowheads). **b** On sagittal T1 fat-suppressed sequence post-gadolinium administration inhomogeneous synovial enhancement can be seen (arrows) indicating fibrotic areas within the inflamed synovium. There is no effusion. Soft tissue oedema is seen in the calf

**Fig. 8:**
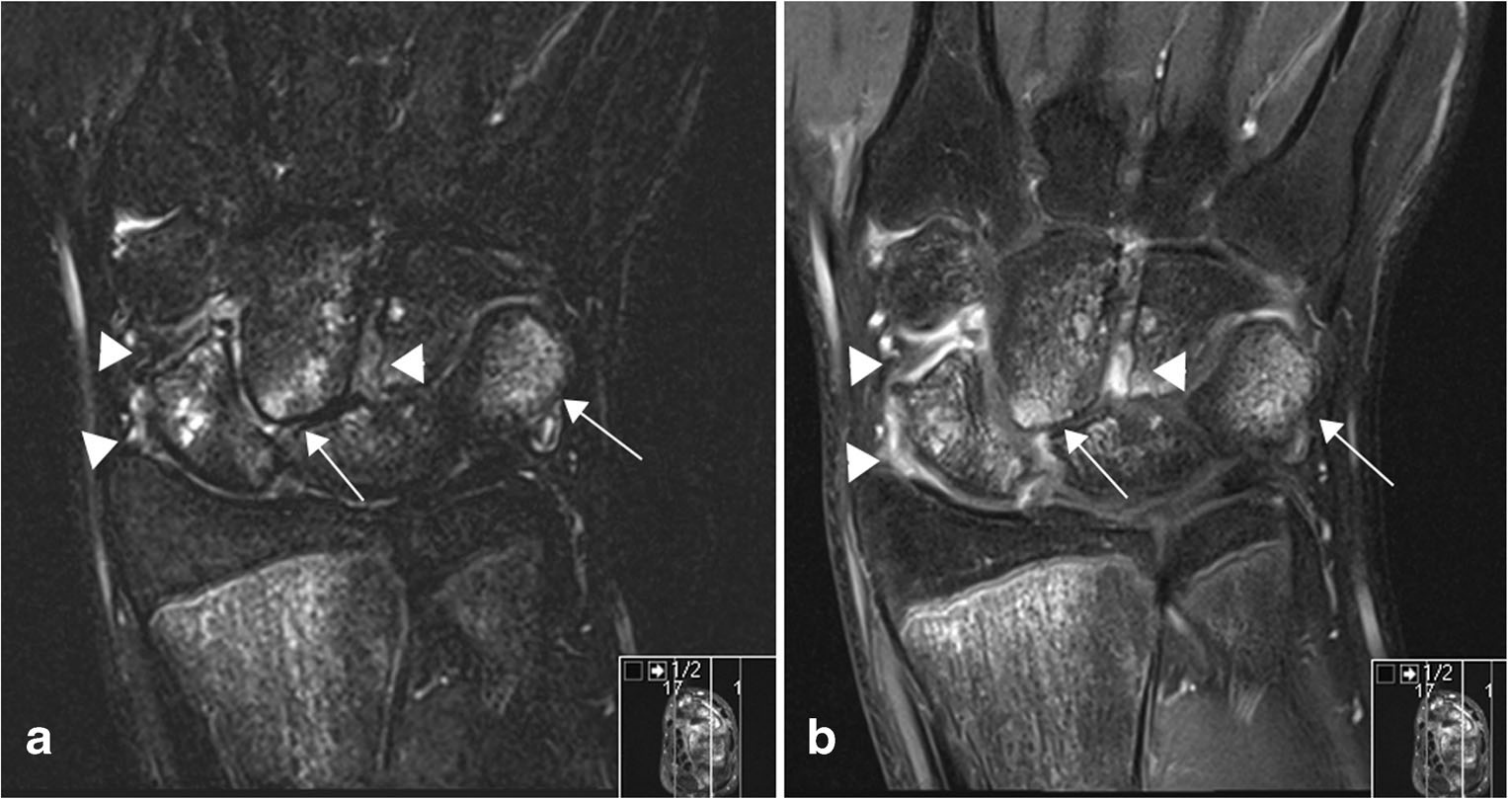
Coronal images of the wrist in a child with active arthritis at the wrist showing high signal within the carpal bones on STIR sequence (**a**), which enhances on the post-contrast T1 f. sequence (**b**), indicative of inflammation (arrows). There is enhancement of a thickened synovium in the intercarpal and radio-carpal joints but no joint fluid (arrowheads)

**Fig. 9:**
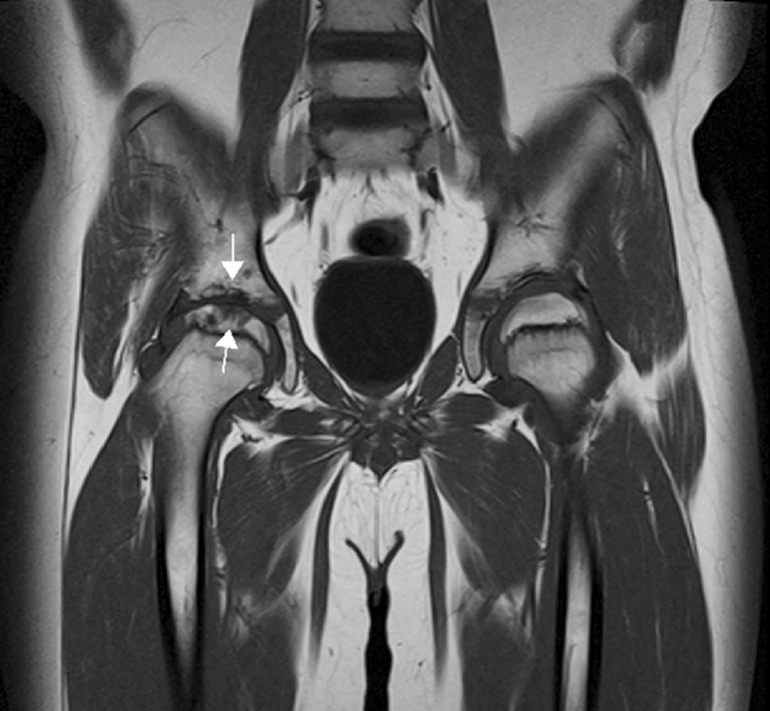
MRI can be used to depict bone destruction. Coronal T1 TSE of the pelvis shows joint space narrowing and erosions of the right hip; the same case as in Fig. 2 (arrows)

In active arthritis there will always be pathological enhancement of a thickened synovium (Figs. 4 and 6). In JIA, as opposed to other types of arthritis, the enhancement may be patchy, particularly in chronic disease, due to areas of fibrotic change (Fig. 7). Increased joint fluid is a common finding (Figs. 4 and 6), but may be absent, particularly in small joints such as the wrist, because of compression by the hypertrophic synovium. Sparse fluid may also be seen in larger joints in chronic synovitis where the synovium is thickened and partly fibrotic (Figs. 7–8). Bone marrow oedema (BMO) in the adjacent skeleton can be caused by reactive osteitis (Fig. 8). Soft tissue oedema and reactive lymph nodes may also be present in long-standing disease or aggressive forms of JIA (Fig. 7). Erosions and/or malalignment of the joint is also depicted on MRI (Fig. 9) and initial studies suggest that MRI has a higher sensitivity to detect erosions than does radiography [18].

**Fig. 10:**
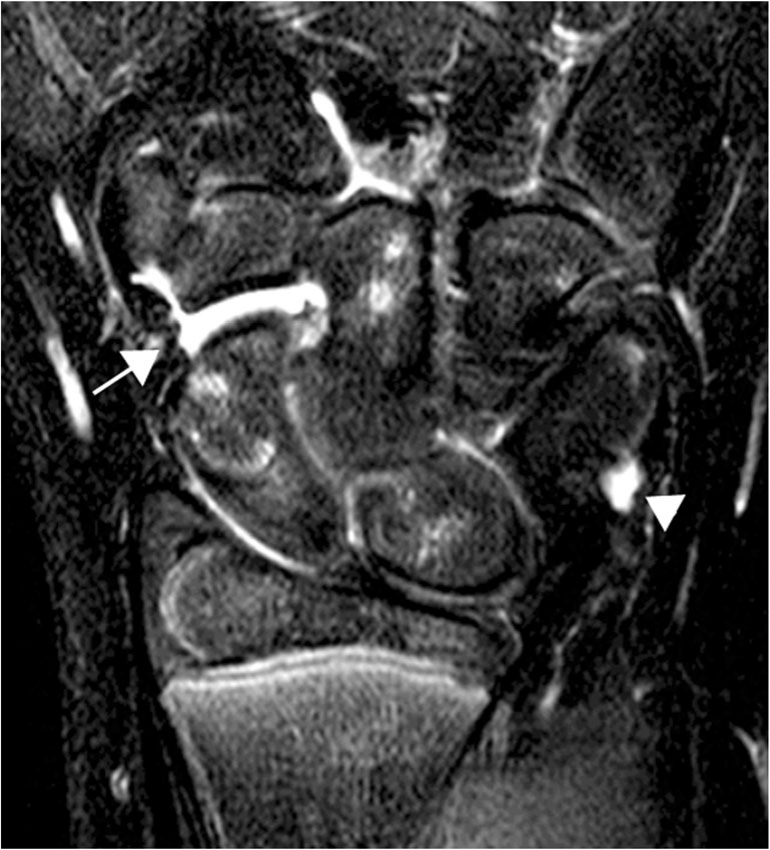
In adults more than 2 mm of joint fluid is defined as pathologically increased fluid at the wrist. In children this is frequently seen as a normal finding (arrow). Fluid in the pisotriquetral recess is also often seen in healthy children (arrowhead), here seen on a coronal STIR of a 12-year-old healthy, asymptomatic boy

**Fig. 11:**
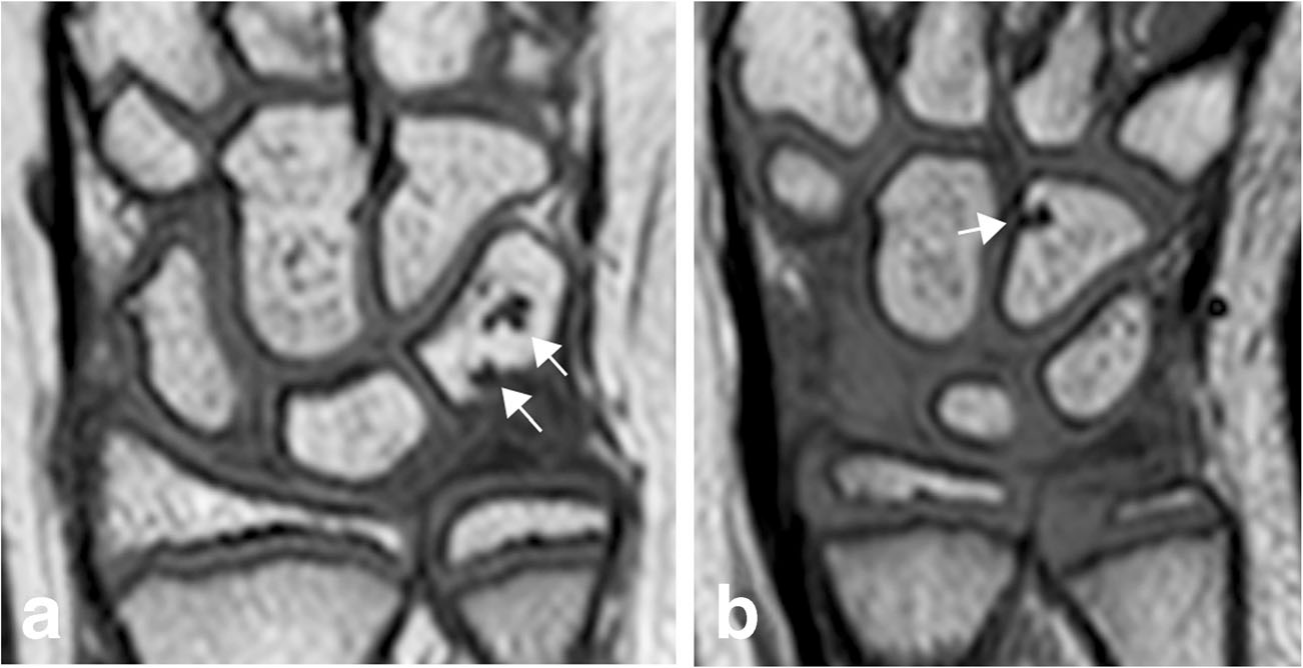
Bony depressions of the carpal bones and proximal metacarpals (arrows) are a normal finding in children and should not automatically be interpreted as erosions. **a** Coronal T1-weighted image of a 10-year-old healthy girl showing typical normal carpal depressions in the triquetrum. In some areas of the carpal bones bony depressions are more frequently seen in healthy children, e.g. in the radial aspect of the hamate, here shown in an 8-year-old, healthy and asymptomatic girl (**b**)

**Fig. 12:**
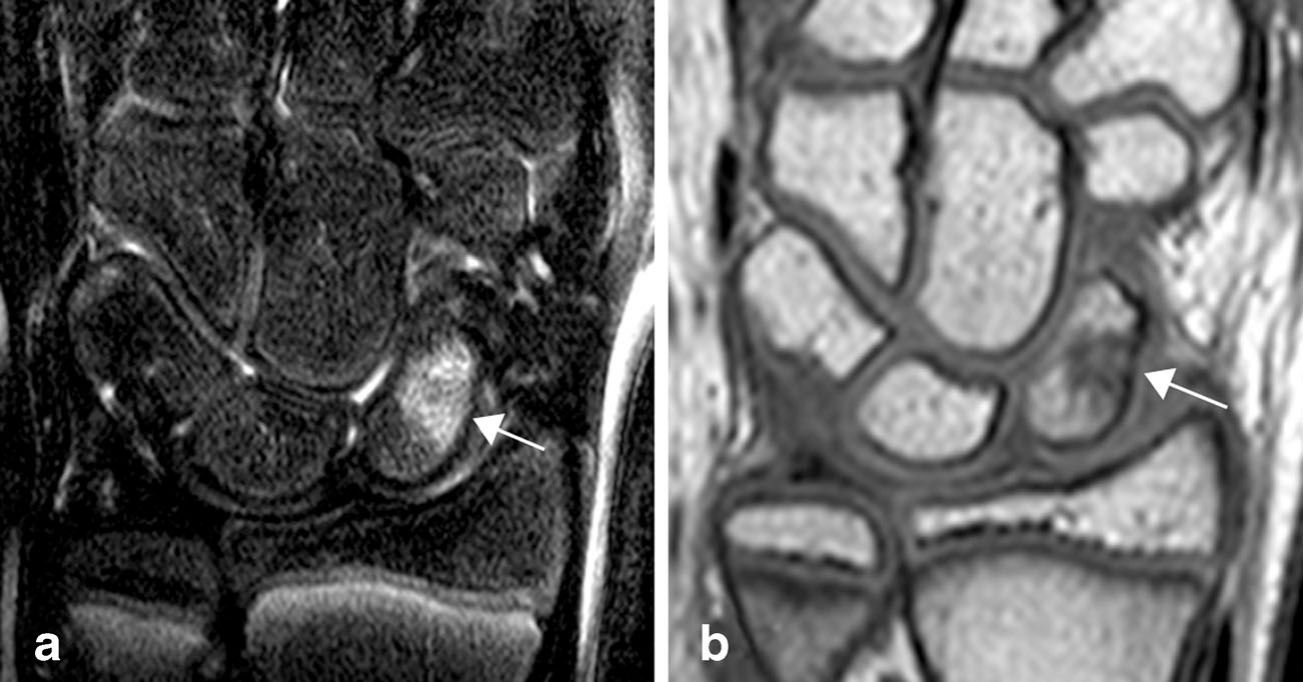
High signal on T2 f. or STIR images (**a**) with corresponding low signal on the T1 TSE sequence (**b**) in the scaphoid suggestive of marrow oedema in a 12-year-old, healthy, asymptomatic boy. Signal changes suggestive of bone marrow oedema can frequently be seen at the wrist in asymptomatic, healthy children

Reading of the MRI examinations in children with arthritis may however be difficult, particularly in cases of subtle findings. Recent studies have shown that lesions suggestive of BMO and amounts of joint fluid that in adults would have been defined as pathological, and changes that look like erosions, are frequently seen on MRI of the wrist also in healthy children [21] (Figs. 10, 11 and 12). To date there is no imaging method that can reliably differentiate these normal findings from those caused by disease. There are some studies describing the normal anatomy and contrast enhancement pattern of the temporomandibular joints [22, 23]; otherwise, there is a paucity of age-dependent normal anatomy on MRI. Additional disadvantages of MRI may be the low availability, high costs and the need for sedation in children younger than 4–6 years.

### CT

Cone-beam computed tomography (CBCT) is currently accepted as the imaging modality of choice for assessment of permanent bony changes to the TMJs. It can visualise joint space narrowing and erosive change, as well as growth disturbances with a relatively low radiation dose [7].

Computed tomography (CT) may be helpful in the assessment of the facet joints, in particular in the cervical spine. Its use is, however, limited by the relatively high radiation dose.

## Validation of imaging markers for active disease and permanent joint damage

There have been major advances in the treatment of JIA during the last decade. The development of new therapeutic agents and new, individually tailored treatment strategies has led to significant improvement in functional outcome in children with JIA [24, 25]. The paramount goal of current treatment in JIA is to achieve inactive disease and remission with or without medication [3]. In order to evaluate the therapeutic response, sensitive and reproducible tools for assessment of change in the severity of inflammation and for bony destruction have become crucial. Of the diagnostic tools currently available, imaging studies are thought best suited for these purposes [26]. The radiological investigations in JIA should ideally be able to determine the presence and degree of (1) active inflammation, (2) precursors of permanent articular damage, (3) established articular damage and (4) complications to treatment.

Synovial contrast enhancement, bone marrow oedema (BMO) and increased joint fluid are all thought to be signs of active inflammation. The presence of bone erosions is a sign of structural joint damage and the presence of BMO may be predictive of later bone erosions [27, 28]. These definitions of active inflammation and destruction are based on research on patients with rheumatoid arthritis and have been adapted for use in children. Recent studies have shown that definitions extrapolated from research in the adult population cannot automatically be used in children and may lead to both over- and under-staging of disease [21, 29–31]. Therefore separate validation of the different modalities when used in children must be performed in order to provide systems that can reliably quantify the degree of inflammation and destruction in JIA. Large efforts have been made over the last decade to provide objective imaging tools for the quantification of disease activity in children with JIA. A summary of the current status is provided below:

### Radiographs

Radiographic scores are only feasible for structural change and not for inflammation. Radiographic scoring systems specific for joint destruction at the wrist in JIA have been devised and show good reproducibility and clinical validity [11]. One initial validation of a scoring system for chronic changes at the hip has been published [32] but validated radiographic scoring systems for other joints are lacking.

### Ultrasound

Apart from studies of the age-dependent cartilage thickness [33], normal appearances for the various joints in a growing child throughout childhood are not well documented on ultrasound. In addition, to date there are no unifying definitions of synovial thickening, pathological amounts of joint fluid, hyperaemia or destructive change on ultrasound; hence, no validated scoring systems for disease activity and progression are available and the interpretation of the US findings in JIA remains subjective [12, 34].

### MRI

Great efforts have been made by different national and international study groups, e.g. the Health-e-Child Radiology Group and the Outcome Measures in Rheumatology Clinical Trials (OMERACT) Special Interest Group ‘MRI in JIA’ to create and validate a universal paediatric MRI scoring system [35]. This work has shown that in multi-centre studies the assessment and semi-quantification of BMO and tenosynovitis have acceptable repeatability for clinical use, whereas synovial enhancement and overall inflammation can be accurately scored by the same observer while inter-observer agreement was less favourable, but acceptable for the assessment of the radio-ulnar and radio-carpal joints [36, 37]. Moreover, recent studies showing that many of the findings that were previously defined as pathology also appear in healthy children have altered the whole perception of the accuracy of the MRI features included in previous scoring systems and the main challenge we now face is how to differentiate normal findings from disease. Bone erosion is the only feature of destruction included in the suggested scoring systems for JIA on MRI. However, it has previously been suggested, when using radiographs in the assessment, that bone erosions may not even be the most significant sign of bone destruction at the wrist, particularly in younger children, and that carpal crowding or squaring of the carpal bones causing a small and deformed wrist is more characteristic for JIA [10, 38]. This was later confirmed in a study comparing healthy children and those with JIA showing that children with JIA had significantly smaller wrists, but there was no difference in the number of erosion-like changes [30]. Including erosions only in the assessment of bone destruction in children may therefore lead to both over- and under-staging of disease. There is ongoing multinational collaboration among several research groups to create accurate and reliable scoring systems for JIA, for both large and small joints, but to date an internationally validated and accepted scoring system for JIA is not yet available for any of the joints on MRI.

## Conclusion

Imaging is a useful diagnostic supplement to the clinical assessment of children with JIA. Following the introduction of new, potent biologic drugs, there is an urgent need for accurate markers of disease, both active arthritis and permanent joint damage. Imaging, in particular MRI, has great potential, but more studies addressing the precision and accuracy as well as the clinical validity of the findings should be done.
